# A Computational Study of the S_2_ State in the Oxygen-Evolving Complex of Photosystem II by Electron Paramagnetic Resonance Spectroscopy

**DOI:** 10.3390/molecules26092699

**Published:** 2021-05-04

**Authors:** Bernard Baituti, Sebusi Odisitse

**Affiliations:** Department of Chemical and Forensic Science, Faculty of Science, Botswana International University of Science and Technology, Private Bag 16, Palapye, Botswana; odisitses@biust.ac.bw

**Keywords:** photosystem II, spectrum, *g*4.1 signal, multiline, oxidation, simulation

## Abstract

The S_2_ state produces two basic electron paramagnetic resonance signal types due to the manganese cluster in oxygen-evolving complex, which are influenced by the solvents, and cryoprotectant added to the photosystem II samples. It is presumed that a single manganese center oxidation occurs on S_1_ → S_2_ state transition. The S_2_ state has readily visible multiline and g4.1 electron paramagnetic resonance signals and hence it has been the most studied of all the Kok cycle intermediates due to the ease of experimental preparation and stability. The S_2_ state was studied using electron paramagnetic resonance spectroscopy at X-band frequencies. The aim of this study was to determine the spin states of the g4.1 signal. The multiline signal was observed to arise from a ground state spin ½ centre while the g4.1 signal generated at ≈140 K NIR illumination was proposed to arise from a spin $\frac{5}{2}$ center with rhombic distortion. The ‘ground’ state g4.1 signal was generated solely or by conversion from the multiline. The data analysis methods used involved numerical simulations of the experimental spectra on relevant models of the oxygen-evolving complex cluster. A strong focus in this paper was on the ‘ground’ state g4.1 signal, whether it is a rhombic $\frac{5}{2}$ spin state signal or an axial $\frac{3}{2}$ spin state signal. The data supported an X-band CW-EPR-generated g4.1 signal as originating from a near rhombic spin 5/2 of the S_2_ state of the PSII manganese cluster.

## 1. Introduction

The invention of photosynthetic oxygen evolution in 4.6 billion years of the history of the earth allowed for the development of oxygenic atmosphere that forms the basis of life activities. Green plants, algae, and cyanobacteria powered by solar energy convert carbon dioxide and water into molecular oxygen and organic matter. This process is catalyzed by a huge membrane pigment–protein complex enzyme in photosystem II (PSII) [1]. The core machinery for oxygen evolution is Mn_4_CaO_5_ cluster (catalytic site) located in photosystem II (PS II) protein complex [2]. The oxygen evolution proceeds, removing four electrons and four protons (H^+^) from the two substrate water molecules at the Mn_4_CaO_5_ cluster of the oxygen-evolving complex (OEC) of PSII [3,4]. The two ligand water molecules, W1 and W2, are located at the dangling Mn4 site in the Mn_4_CaO_5_ cluster, while another two water ligands (W3 and W4) are located at the Ca^2+^ site [5]. The structure of the multi-sub-unit pigment–protein complex (PSII) has been studied extensively by X-ray diffraction (XRD), with a resolution ranging from 3.8 to 1.9 Å using synchrotron radiation (SR) X-ray sources [6,7,8,9,10,11]. The structure of PSII from different photosynthetic organisms has been revealed by both cryoelectron microscopy and X-ray crystallography [2,7,12,13,14,15]. The PSII electron transfer process is divided into ‘acceptor’ and ‘donor’ sides. The acceptor side receives an electron from the reduced P_680_*, hence oxidizing it to P_680_^+^ (estimated reduction potential of 1.2 V). The P_680_^+^ formed is reduced from the ‘donor’ side by oxidizing the Mn_4_CaO_5_ cluster [16]. The OEC is oxidized successively by P_680_^+^ four times, then resets itself to the most reduced state after it converts water into molecular oxygen. The redox-active tyrosine residue of D1 subunit called tyrosine ‘Z’ (Tyr_Z_) electrically links the OEC to P_680_ [17,18,19]. The catalytic mechanism of the OEC is still unresolved. The oxidation states of the Mn ions and how they change along the catalytic site is not clear. The manganese cluster undergoes four successive oxidations, progressing through a series of different net valence states (so-called S_i_ states, where i = 0–4, i denotes the stored oxidizing equivalents). The oxidation state of the OEC, S_i_, increases as electron transfer occurs. Oxygen evolved in the $S_{3}\to S_{0}$ transition while proton release is noticed with stoichiometry 1:0:1:2 for $S_{0}\to S_{1}\to S_{2}\to S_{3}\to S_{0}$ [5]. The S_0_ and S_2_ states of the catalytic site have a net unpaired spin ½ arising from anti-ferromagnetic couplings of the Mn ions, which makes them paramagnetic and thus can be studied using electron paramagnetic resonance (EPR) spectroscopy. The S_2_ (and S_0_) states yield Mn-derived hyperfine structured, so-called multiline (ML) signals (included over 20 resolved lines hence called ‘multiline’), centered on g ≈ 2 at low temperatures [20,21,22]. The Mn ions are exchange coupled in all Kok cycle S-states [23]. This is mainly anti-ferromagnetic—the spins oppose each other. S_0_ and S_2_ have a net spin ½ ground state. This effective single electron spin interacts with all Mn nuclei (each Mn ion has nuclear spin $I=\frac{5}{2}$), giving rise to a nuclear hyperfine (HF)-structured ML pattern through the nuclear HF couplings $A_{i}$. In the exchange-coupled system, these are given by $A_{i}=\rho_{i}A_{i\left(\mathrm{ion}\right)}$, where $A_{i\left(\mathrm{ion}\right)}$ is ‘isolated ion’ HF value. The projection coefficients are dimensionless and sum 1 for the whole Mn cluster [24]. An estimate of this HF value can be obtained from a single determinant quantum chemical calculation on the geometry-optimized S_2_ state Mn cluster [25]. The $\rho_{i}$ (projection of the total spin onto ${\mathrm{Mn}}_{i}$) is the projection coefficient arising from the coupling in the system within the S_T_ = ½ manifold. Since the OEC is a sufficiently strongly exchanged coupled system, it is therefore the effective Mn HF interaction’s ‘spin projected’ values that are seen, not the intrinsic ‘single’ ion value. The spin projections reflect the contribution of each Mn ion to a total spin state. The pulsed electron–electron double resonance (PELDOR) measurements carried out between the S_2_ ML signal and Try_D_ (situated ≈30 Å from the OEC) radical dipole/dipole interaction are consistent with the data of Jin et al. [25] and Kurashinge et al. [26], with *ρ*_1_ ≈ 2, *ρ*_2_ ≈ −1.2, *ρ*_3_ + *ρ*_4_ ≈ 0.2 [27]. Therefore, the HF couplings seen from the Mn^III^ is ≈ twice that from Mn^IV^ and much more anisotropic. Since the dominant line spacing of the ML signal is ≈90 G (250 MHz) and the width of the ML signal is ≈1800 G (5.5 GHz), it means that one coupling must be large since two Mn couplings are small. This is because, in first order

ML width (1800 G) ~ 5(A_1eff_ + A_2 eff_ + A_3 eff_ + A_4 eff_) (1)

Here, the A_i eff_ are effective, ≈angularly averaged HF values. The $\rho_{i}$ can be described as a measure of electron density of each Mn ion in the cluster or describe the contribution of each of the Mn ions in the Mn_4_CaO_5_ cluster to a total spin state. All the four Mn contribute to the ML signal through its HF interactions in the OEC. The total width is determined by the contributions from individual Mn ions. The effective spin Hamiltonian describing ground state S_T_ = ½ is

(2) $$H=g_{S}\beta \cdot S_{T}\cdot H_{z}+\sum_{1}^{4}Ii\cdot a_{i}\cdot \mathrm{ST}+\sum_{1}^{4}I_{i}\cdot Q_{i}\cdot I_{i}$$

The first term in Equation (2) represents the total electron Zeeman term, while the second term represents HF terms, and the last term the nuclear quadrupole.

Casey et al. [28], discovered another S_2_ state signal formed by ≈140 K illumination of PSII samples, also attributed to the manganese cluster and called the g4.1 signal, centered near g ≈ 4.10, which can appear with or without the presence of the ML signal [29]. Following its initial discovery by Casey et al., it was subsequently shown that the ML signal to g4.1 inter-conversion could be stimulated by near infrared (NIR) illumination at about 140 K in PSII centers already in the S_2_ state [30]. It is likely that some NIR was present in the illuminations originally used by Casey et al. The ‘ground’ state g4.1 signal has been proposed to arise from a rhombic spin $\frac{5}{2}$ center [31] or near axial spin $\frac{3}{2}$ center [32].

**Figure 1:**
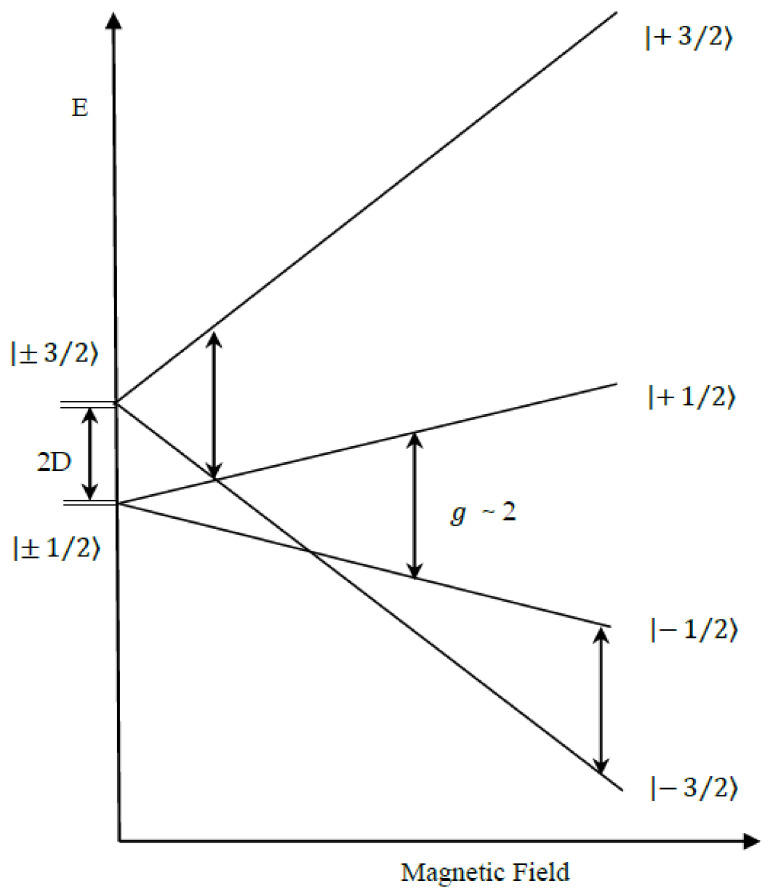
The axial spin $\frac{3}{2}\;$ state ZFS and the Zeeman splitting when the field is parallel to the molecular axis. The arrows indicate the microwave quantum energy *E = hν*. Figure based on a diagram provided in Carrington et al. [33].

A strong focus in this paper has been on the ‘ground’ state g4.1 signal, whether it is a rhombic $\frac{5}{2}$ spin state signal or an axial $\frac{3}{2}$ spin state signal. A quartet state is when an ion has three unpaired electrons with the total spin of $\frac{3}{2}$ (see Figure 1). This state with an odd number of electrons is a Kramers system and the electronic states are at least doubly degenerate (±$\frac{1}{2}$ and ±$\frac{3}{2}$) in the absence of external magnetic fields. These Kramers doublets are degenerate states for any molecule with an odd number of electrons (±$\frac{1}{2}$, ±$\frac{3}{2}$, ±$\frac{5}{2}$, ±$\frac{7}{2}\ldots .$). The zero-field splitting’s (ZFS) and deviation from regular symmetry produce two Kramers doublets: *m_s_* = ±$\frac{1}{2}$ and *m_s_* = ±$\frac{3}{2}$. The Zeeman splittings also depend on the orientation of the magnetic field with respect to the molecule. The spin Hamiltonian for the EPR spectra becomes [33]

(3) $$H=\beta H\cdot g\cdot Ŝ\;+\;D[{Ŝ}_{z}^{2}\;-\frac{5}{4}]\;+E\;\left[{Ŝ}_{x}^{2}-{Ŝ}_{y}^{2}\right]$$

The first term in the Hamiltonian (Equation (3)) represents the electron Zeeman term, while the second term is the ZFS interaction, with *E* = 0 for axially symmetric. The degeneracy of the Kramers doublets is removed by the magnetic field along the external *z* direction. The energy gap of the splitting is 2D (Figure 1) between the two states, which are Kramers pairs, with D representing the energy of ZFS. These degenerate doublets undergo splitting when an external magnetic field is applied.

**Figure 2:**
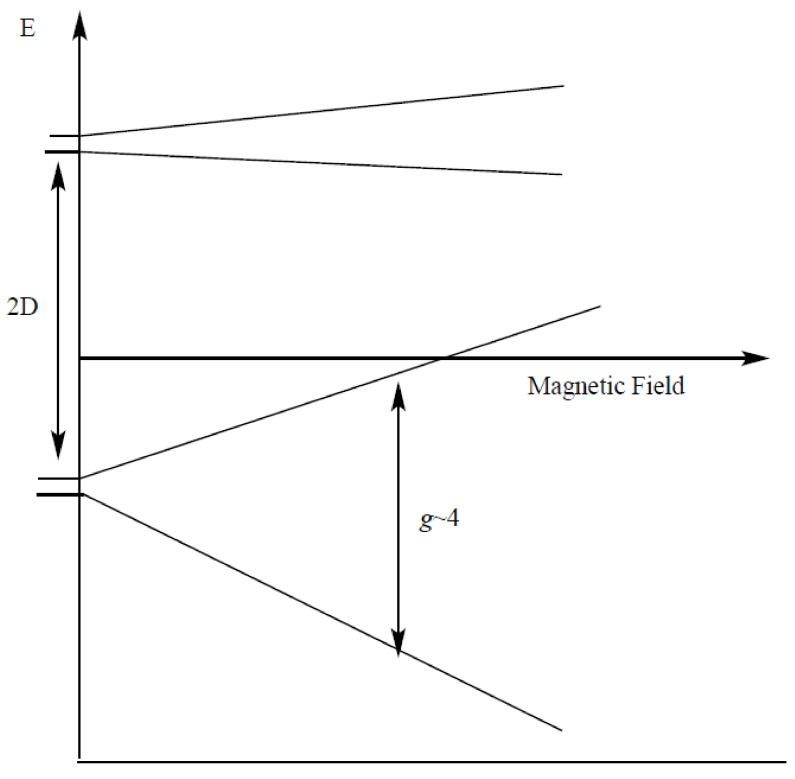
The axial spin $\frac{3}{2}\;$ state ZFS and the Zeeman splitting when the field is perpendicular to the molecular axis.

If the ZFS is greater than the applied microwave quantum (E=hv), then the transition $+\frac{1}{2}↔+\frac{3}{2}$ will not be accessible, while the lower one ($-\frac{1}{2}↔-\frac{3}{2}$) will require very high magnetic fields to be observed. These leave $-\frac{1}{2}↔+\frac{1}{2}$ as the only transition that can be observed under any amount of ZFS. Therefore, only a g≈ 2 transition will be visible if the parallel external magnetic field is applied parallel to the molecular ZFS axis (Figure 1). If the external magnetic field is applied perpendicular (Figure 2) to the molecular symmetry axis, the splitting’s of the Kramers doublets will be different from when the field is applied parallel due to the Zeeman interaction. Here, the states $\pm \frac{1}{2}$ and $\pm \frac{3}{2}$ mix instead of forming the simple Kramers doublets, forming new states with combination of both the $\frac{1}{2}$ and $\frac{3}{2}$ states, which splits differently from the earlier case during the Zeeman interaction. The transitions occur around g = 4 and the g4.1 peak will be visible (Figure 2).

An ion with five unpaired d electrons (high spin) has total spin = $\frac{5}{2}$, and the *m_s_* can be $+\frac{5}{2}$, $+\frac{3}{2}$, $+\frac{1}{2}$, $-\frac{1}{2}$, $-\frac{3}{2}$, $-\frac{5}{2}$. These electrons, in both tetrahedral and octahedral symmetry, occupy the d orbitals as follows: two occupy the degenerate *e_g_* orbitals while the remaining three occupy the triply degenerate *t*_2*g*_ orbitals. This means the ground state orbitals are nondegenerate, and any excited state will involve the promotion of an electron from *t*_2*g*_ to *e_g_* orbitals or from *e_g_* to *t*_2*g*_ orbitals. Since the orbitals are non-degenerate, as opposed to the quartet state above, then spin orbit coupling will be negligible and hence the ZFS would be quite small. Nonetheless the spin degeneracy is still removed in this kind of complex, with direct electron dipole spin–spin couplings or higher order spin–orbit perturbations modifying the spin Hamiltonian as follows [33]:

(4) $$H=g\beta H\cdot Ŝ+\frac{1}{6}a[{Ŝ}_{x}^{4}+{Ŝ}_{y}^{4}+{Ŝ}_{z}^{4}-\frac{707}{16}]$$

The first term in the Hamiltonian (Equation (4)) represents the electronic Zeeman term, while the second term represents the direct electron dipole spin–spin couplings or higher order spin–orbit perturbations. Ŝ is the fictitious spin. This results in the splitting of the rhombic $\frac{5}{2}$ spin state into three Kramers doublets (*m_s_* = ±$\frac{1}{2}$, *m_s_* = ±$\frac{3}{2}$, and *m_s_* = ±$\frac{5}{2}$). The three Kramers doublets have three groups of states, with all the transitions except $-\frac{1}{2}↔+\frac{1}{2}$ with g = 2 being forbidden by ∆*m_s_* = ±1 selection rule. The degeneracy is removed when a magnetic field is applied, with the splitting produced proportional to the applied magnetic field because it occurs in first order perturbation [34].

**Figure 3:**
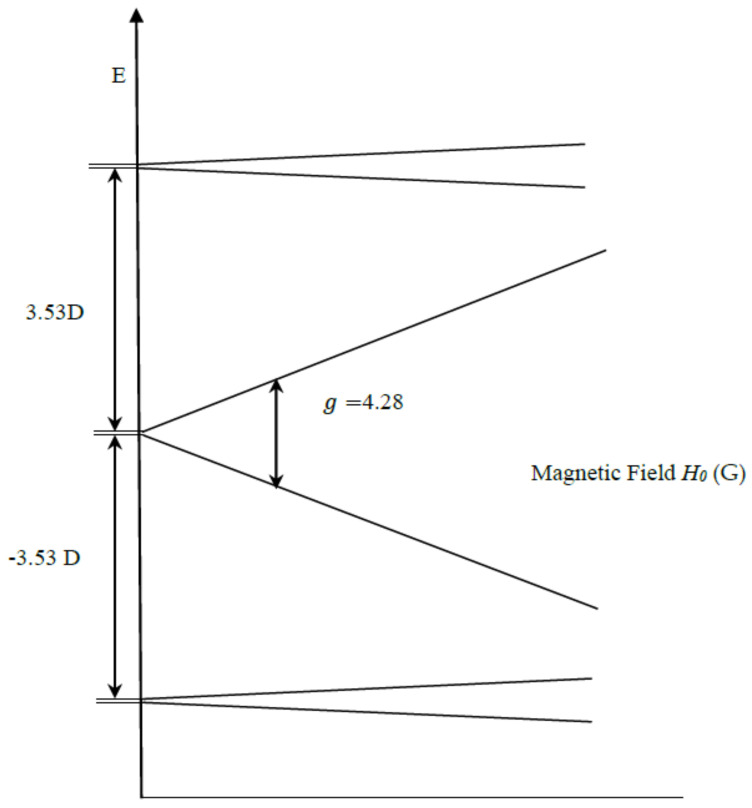
Illustrates the pure rhombic spin $\frac{5}{2}$ ZFS and Zeeman splitting.

For a pure rhombic system, only the terms ${Ŝ}_{x}^{2}-{Ŝ}_{y}^{2}$ are significant in ZFS, and the three Kramers doublets are once again split into three degenerate pairs. The pair with splitting energy W = 0 (Figure 3) is characterized by g = 4.286, which is independent of orientation, i.e., isotropic [34]. The two other pairs (upper and lower) have anisotropic (dependent on orientation) transitions, with g = 9.678, 0.857, and 0.607 values [34]. Figure 3 below shows the energy level-splitting diagram associated with the pure rhombic spin $\frac{5}{2}$- state. This is characterized by the quasi-isotropic EPR transition at g≈4.2−4.3. This spin species is examined here as the possible basis for OEC resonances in the S_2_ state.

## 2. Results and Discussion

The difference spectra for the ML signals, and g4.1 and ‘g2’ NIR signals were obtained by subtracting the appropriate background, or pre-NIR illumination spectra, from the ≈140 K NIR illuminated spectra.

**Figure 4:**
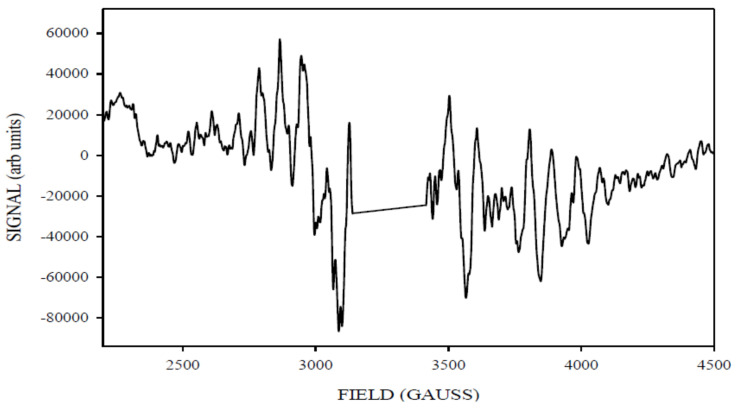
The X-band CW-EPR-generated difference ML signal obtained corresponding to light (≈140 K NIR illumination) minus annealed spectra. Spectrometer conditions: 15 G modulation amplitude, center field 3400 G, sweep width 2500 G, 0.5 mW microwave power, frequency 9.369126 GHz, temperature 6 K, number of points 2500.

**Figure 5:**
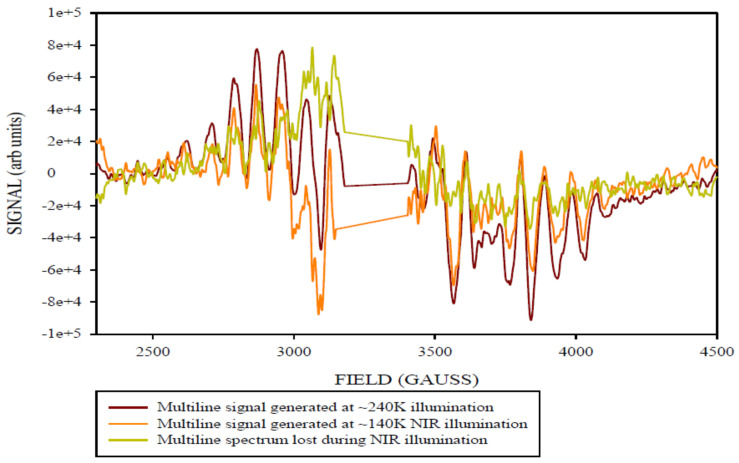
The X-band CW-EPR-generated ML signal obtained (≈240 K difference) minus (≈140 K NIR difference) spectra. This corresponds to the amount of ML signal lost (≈35%) when illuminating the sample at a lower temperature of ≈140 K using the NIR light.

The ML signal (Figure 4) that resulted exhibited even more HF structured detail compared to the ML signal generated from the ≈240 K illumination. This suggests that the ML signal generated by ≈240 K illumination here (and likely generally) is not a strictly uniform species with regards to factors contributing to fine spectral detail. The resultant spectrum obtained in Figure 5 was obtained after subtracting the ≈140 K NIR ML difference spectrum from ≈240 K ML difference spectrum, which represents the amount of ML signal lost (≈35%) when re-illuminating the sample at ≈140 K with NIR light to photo-induce the g4.1 signal. It is evident from the two spectra that the ML signal generated at ≈240 K illumination had more overall intensity than the one remaining after ≈140 K NIR illumination. This suggests that the g4.1 signal (be it ‘ground’ or ‘excited’ state) was formed at low temperature illumination (approximately ≈140 K) by inter-conversion of a sub population of S_2_ ML centers. Re-conversion back to ML signal centers occurs by dark adaptation at higher temperatures, ≈240 K for PSII core samples and 200 K for PS II membrane samples [29]. This is all consistent with the original observation of ML signal to g4.1 inter-conversion stimulated by near infrared illumination at about 140 K temperatures in PSII membrane centers [30]. The procedure was repeated at several microwave powers of (0.2 mW, 1 mW) at 6 K in order to check for power saturation effects, and at a range of temperatures (5, 8, 15, and 20 K, with other parameters held constant). The ML signals generated at various temperatures showed some differences in the signal intensity (beyond expected Curie effects), with the maximum signal intensity observed at 6 K, with some decrease of the signal intensity at 5 K. The origin(s) of these effects, if real, was not pursued further here.

The 140 K NIR illuminated-minus-annealed spectrum for PSIINoAdds core sample.

**Figure 6:**
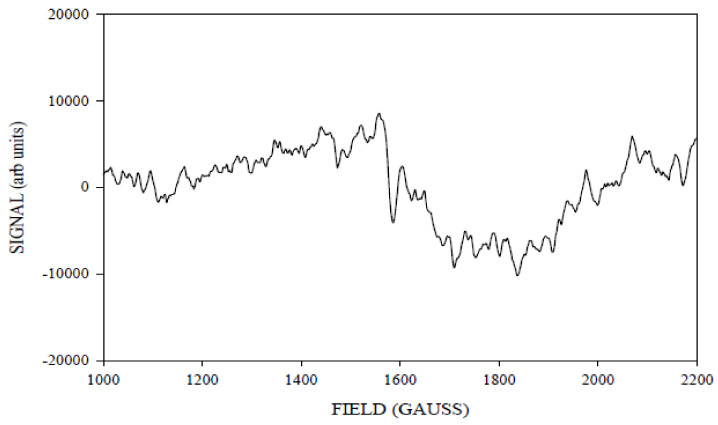
The X-band CW-EPR difference g4.1 spectrum obtained corresponding to the light (≈240 K illumination) minus annealed spectra. Spectrometer conditions: 15 G modulation amplitude, center field 1650 G, sweep width 2500 G, 0.2 mW microwave power, frequency 9.370369 GHz, temperature 6 K, number of points 2500.

Figure 6 shows the ≈240 K green illuminated g4.1 difference spectrum. It was observed between 1000 and 2200 G in the magnetic field axis at X band.

**Figure 7:**
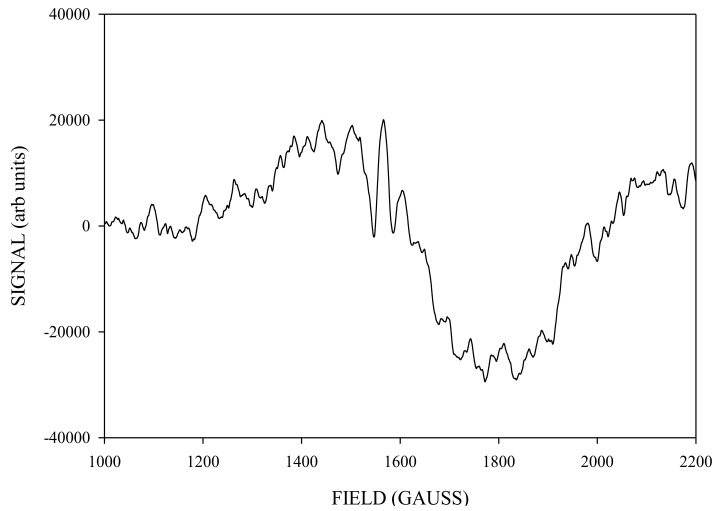
The X-band CW-EPR-generated difference g4.1 signal obtained corresponding to light (≈140 K NIR illumination) minus dark S_1_ annealed spectra. Spectrometer conditions: 15 G modulation amplitude, center field 1650 G, sweep width 2500 G, 0.2 mW microwave power, frequency 9.370369 GHz, temperature 6 K, number of points 2500.

**Figure 8:**
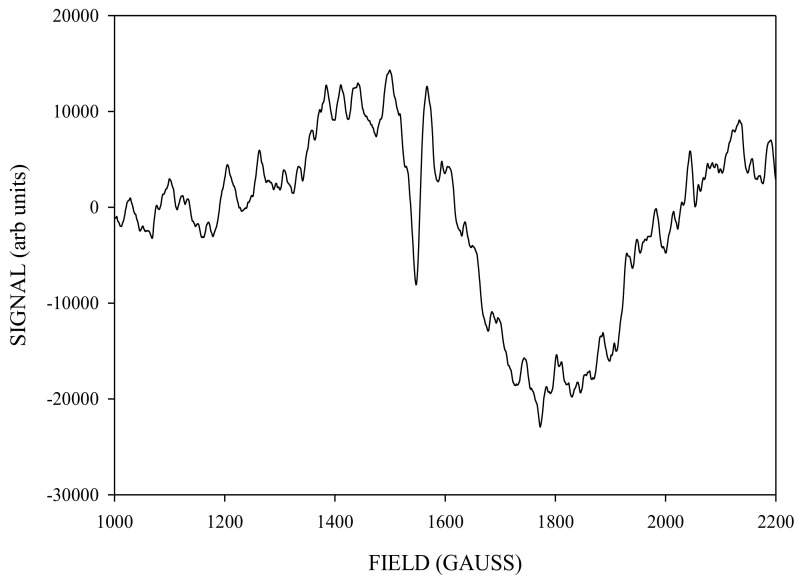
The X-band CW-EPR difference g4.1 spectrum obtained corresponding to Figure 7 (140 K NIR difference) minus Figure 6 (≈65% of 240 K difference) spectra.

To isolate the ‘pure’ spectrum of the g4.1 species generated by NIR turnover, we subtracted from the 140 K NIR-illuminated g4.1 (Figure 7) an appropriately scaled amount of the 240 K green illuminated g4.1 (Figure 6, about 65% of the signal that did not interconvert) to obtained the spectrum in Figure 8.

### Simulation of X-Band CW-EPR-Generated g4.1 Signal of ≈140 K NIR-Illuminated PSII Core Samples

**Figure 9:**
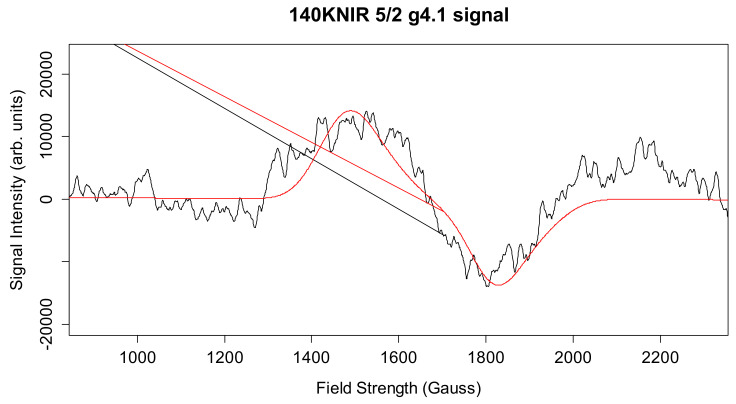
The experimental X-band CW-EPR difference g4.1 spectrum and best-fit simulation as a rhombic spin 5/2 state (red spectrum, see Table 1). Spectrometer conditions: center field: 1650 G; frequency: 9.369115 GHz; microwave power 0.2 mW; sweep width: 2500 G; number of points 2500; number of scans: 5; temperature: 8 K; modulation amplitude 15 G (see Supplementary Materials S1).

**Figure 10:**
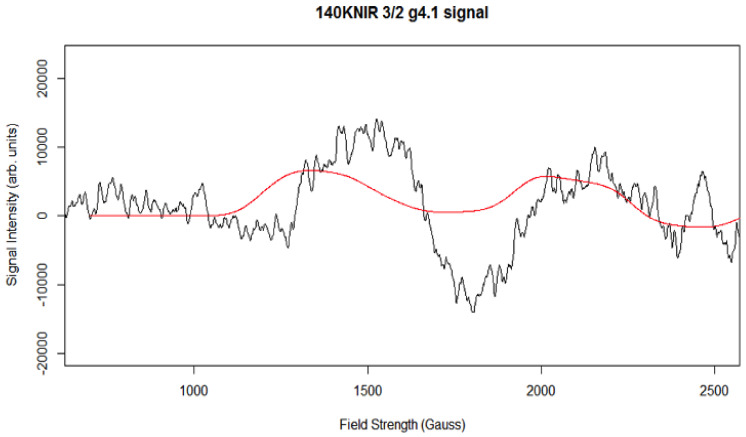
The experimental X-band CW-EPR difference g4.1 spectrum and best-fit simulation (red spectrum). Spectrometer conditions: center field: 1650 G; frequency: 9.369115 GHz; microwave power 0.2 mW; sweep width: 2500 G; number of points 2500; number of scans: 5; temperature: 8 K; modulation amplitude 15 G (see Supplementary Materials S2).

The g4.1 signal experimental spectrum together with best fit simulation as a 5/2 state is shown in Figure 9. Table 1 lists the simulation parameters; the matrix diagonalization method was used. Figure 9 shows the best fit simulation, which suggests that g4.1 signal may originate from the spin 5/2 state. This is unlikely to be strictly accurate but given that no HF structure is resolved on any of the excited state, spin > ½ signals, it is a reasonable, minimal assumption. No plausible fit to the g4.1 signal could be found with rhombic symmetry and a spin 3/2 state, but interestingly Figure 10 shows that such a species may have been present, with one predicted up-field feature around 2200 G and the other possibly overlapping with the spin 5/2 g4.1 signal. Thus, the spin ½ and spin 3/2 states expected to accompany the easily visible spin 5/2 state arising from NIR turnover may have been present. It is a unique feature of the spin 5/2 system in rhombic symmetry that all three principal axis transitions were near co-incident at X band, making this signal easily seen. For other spin states in the manifold of this system, the principal axis transitions were generally well separated in the EPR spectrum and harder to identify (and thus quantitate). Hence, the data support an X-band CW-EPR-generated g4.1 signal as originating from a near rhombic spin 5/2 of the S_2_ state of the PSII manganese cluster. Therefore, the conclusion that can be drawn from the ≈140 K NIR-illuminated PSII core samples is that, in addition to a clear rhombic spin 5/2 state, the NIR turnover likely generated a g≈2 signal (S = 1/2) as indicated in Figure 5 and a spin 3/2 system (Figure 10), although temperature dependence studies of the latter two were not performed.

## 3. Materials and Methods

The PSII membranes and PSII core samples used were obtained from fresh market spinach, commonly known as English spinach (*Spinacia oleracea*). The protocol used in this project in isolating the PSII containing thylakoid membranes is by Bricker et al. [35], with modification by Smith et al. [36]. The homogenization and incubation steps as well as centrifugation were carried out in a dim green light cold room, with temperature of about 4 °C. The light harvesting complex II (LHC II) contains almost 80% chlorophyll in the PSII membrane, and the separation of this LHC II results in isolating the PSII core complex, which is embedded inside the larger PSII assembly. Isolation of the PSII core and reaction center (RC) was performed following the protocol by Van Leeuwen et al. [37] with modifications by Smith et al. [38].

### 3.1. Illumination and Annealing Procedures

The PSII samples prepared were thawed on ice/water mix (0 °C/273 K) for 1 h before use. Aliquots of 250 µL were carefully loaded into 4 mm O.D. quartz EPR tubes (Wilmad quartz) and subsequently frozen in liquid nitrogen. If bubbles were found in the sample, it was immediately thawed, the bubble was removed, and the sample was then re-frozen in liquid nitrogen (about 77 K). The annealing of PSII sample was performed by storing the sample in ice/water mix (0 °C/273 K) covered with a black cloth to minimize stray light entering. The sample was annealed for approximately 10 to 30 min, ensuring a full relaxation to the S_1_ state, and then the sample was subsequently freeze trapped in the S_1_ state using liquid nitrogen. The S_1_ state generates the background EPR spectrum, which was subtracted from an illumination spectrum to achieve the difference spectrum. The tyrosine radical (Y_D_) signal was also removed from the difference spectrum. Otherwise, the remaining tyrosine radical signal was subtracted using the Bruker WIN EPR system software package.

To generate the S_2_ state signal as well as the inter-conversion of the ML signal to the ‘ground’ state g4.1 signal, we subjected samples to continuous illumination at specific temperatures and wavelengths of light. To generate the S_2_ state thorough S_1_ → S_2_ turnover, from PSII core complexes or PSII membranes, the temperature of the sample was firstly monitored and controlled by a nitrogen gas flow system, and then samples were subjected to 12 s illumination (PSII core samples ≈240 K) or 2 min (PSII membrane samples ≈200 K) using a Kodak Ektalite 1500 slide projector fitted with a halogen lamp. The light was passed through a 10 cm water path IR filter and filtered using a combination of yellow and blue filters to allow only the green light to pass. The sample was rapidly freeze-trapped in the S_2_ state immediately after illumination in the liquid nitrogen (77 K).

Generation of the g4.1 signal was achieved through the inter-conversion of the ML signal-S_2_ state to g4.1 signal by cooling the sample to ≈140 K using the nitrogen flow system and NIR illumination. The light source for the NIR illumination was a 200 W slide projector. The far-red light filters RG9 and RG750 were placed in front of the light source to achieve illumination with low wavelength cut-off of 750 nm. This setup ensured that NIR and far-red light passed through the filter and reached the sample. The sample was illuminated for 12 min. The inter-conversion of the g4.1 signal to ML signal was achieved by increasing the temperature to 200 K for PSII membranes and around 240 K for PSII core samples.

### 3.2. X-Band CW-EPR Spectrometer

The experiments were performed using the X-band continuous wave (CW) EPR spectrometer on PSII containing samples (cores/membranes) trapped in the S_1_ (annealed-taken as a background spectrum) and S_2_ states (illuminated spectrum) and inter-conversion of the ML to the g4.1 form of the S_2_ state. The instrument used was a Bruker Biospin ELEXSYS E500 spectrometer equipped with Bruker SHQX resonant cavity and super X-EPR Microwave Bridge. The spectrometer is fitted with an Oxford–ES900 continuous flow helium cryostat, with temperatures controlled using Oxford Instruments ITC-4 temperature controller.

## 4. Conclusions

The *g*4.1 species formed by NIR-induced turnover from the ML signal S_2_ state is again almost certainly a near rhombic spin 5/2 state, as has been previously proposed [28]. The nature of the putative spin ½ state near g ≈ 2 is much harder to identify presently. Typically, NIR stimulation of Mn mixed valence compounds induces intervalence transfer in oxo-bridged systems, i.e., here between Mn III and Mn IV centers [39]. Since Mn2 is strongly indicated from computational chemistry [40] to be the single Mn^IV^ species present in S_2_ (ground state) of the OEC in the low oxidation paradigm (LOP), the NIR excitations could result in exchange of this oxidation level with Mn1 or Mn3. The possible explanation of the ≈140 K NIR-generated signals may be the inter-valence charge transfer between Mn2 and Mn3 or Mn1, and hence there may be a possible S_2_ low oxidation state pattern of III III IV III or IV III III III, respectively, as earlier assumed [25,41,42,43,44,45,46,47].

## Figures and Tables

**Table 1:** Parameters used in *g*4.1 signal (* fine structure terms similar to Haddy et al. 1992 [31]).

	*gx*, *gy*, *gz*	D (cm^−1^)	E/D	Mn1, Mn2 (MHz)	Linewidth (Gauss)	Spin
*g*4.1 signal	2.18, 2.16, 1.98	* 0.45	* 0.25	194.2, 45.2	140	5/2
*g*4.1 signal	2.18, 2.16, 1.98	0.3	0.3	194.2, 45.2	140	3/2

## Data Availability

Not Applicable.

## Supplementary Materials

Simulation parameters for Figure 9 (S1) and simulation parameters for Figure 10 (S2).

## Sample Availability

Samples of the compounds are not available from the authors.
