# Supplementary material for: A Computational Study of the S2 State in the Oxygen-Evolving Complex of Photosystem II by Electron Paramagnetic Resonance Spectroscopy
Source: Molecules. 2021 May 4;26(9):2699. doi: 10.3390/molecules26092699 (PMC8125536; doi:10.3390/molecules26092699)
Supplement: Supplementary file 1 [file molecules-26-02699-s001.zip › molecules-1177817-supplementary.pdf]

A computational study of the S_2 state in the oxygen-evolving complex of photosystem II by Electron Paramagnetic Resonance spectroscopy.

Bernard Baituti & Sebusi Odisitse

Botswana International University of Science and Technology, Private Bag 16, Palapye, Botswana, Tel +2674931541 Fax: +267 4931541 baitutib@biust.ac.bw

S1. g4.1

```
clear all
clc

% fit the 'broad' experimental spectrum
[B,spc]=textread('g4.1_140KNIR.txt','%f %f');

Sys.S = 5/2;
Sys.Nucs = 'Mn,Mn';
Sys.g = [ 2.16 2.18 1.92];
Sys.A = [194.2; 45.2];
Sys.D = [8993.7 2248.425];
Sys.lw = 14;

Exp.mwFreq = 9.375762;
Exp.CenterSweep = [165 250];
Exp.Range = [70 280];
Exp.nPoints = 2100; % no. points

Opt.Method = 'matrix';

[x,y1] = pepper(Sys,Exp,Opt);

domain = [Exp.Range(1):(Exp.Range(2)-Exp.Range(1))/(Exp.nPoints-1):Exp.Range(2)];
outdata = transpose(vertcat(domain, y1))
csvwrite('g4.1_140KNIR_13_pepper.csv', outdata);
```

S2. g4.1

```
clear all
clc

% fit the 'broad' experimental spectrum
[B,spc]=textread('g4.1_140KNIR.txt','%f %f');

Sys.S = 3/2;
Sys.Nucs = 'Mn,Mn';
Sys.g = [ 2.16 2.18 1.92];
Sys.A = [194.2; 45.2];
Sys.D = [8993.7 2698.11];
Sys.lw = 14;

Exp.mwFreq = 9.375762;
Exp.CenterSweep = [165 250];
Exp.Range = [70 280];
Exp.nPoints = 2100; % no. points

Opt.Method = 'matrix';
```

```
[x,y1] = pepper(Sys,Exp,Opt);

domain = [Exp.Range(1):(Exp.Range(2)-Exp.Range(1))/(Exp.nPoints-1):Exp.Range(2)];
outdata = transpose(vertcat(domain, y1))
csvwrite('g4.1_140KNIR_14_pepper.csv', outdata);
```
